# Supplementary material for: Mitigation of Acetamiprid Residue Disruption on Pea Seed Germination by Selenium Nanoparticles and Lentinans
Source: Plants (Basel). 2023 Jul 27;12(15):2781. doi: 10.3390/plants12152781 (PMC10420818; doi:10.3390/plants12152781)
Supplement: Supplementary file 1 [file plants-12-02781-s001.zip › plants-2507052-supplementary.pdf]

Table S1 Gradient elution program of mobile phase for analyzing acetamiprid

Time (min)	Phase A (%)	Phase B (%)	Flow rate (mL/min)
0.00	95.00	5.00	0.200
3.00	70.00	30.00	0.200
5.00	1.00	99.00	0.200
5.50	1.00	99.00	0.200
5.80	95.00	5.00	0.200
6.20	95.00	5.00	0.200

Note: phase A is 0.1% (v/v) formic acid aqueous solution; phase B is acetonitrile.

Table S2 Mass parameters for analyzing acetamiprid concentration

Compound	Retention time (min)	Parent ion (*m/z*)	Daughter ion (*m/z*)	Cone voltage (V)	Collision energy (eV)	Polarity
Acetamiprid	1.42	233.0	126.0*/56.0	80	15/15	+

Note: * indicates quantitative ion.

Table S3 Linear relation of acetamiprid

Matrix	Linear equations	Linear range (mg/L)	R^2	Matrix effect (%)
Acetonitrile	y=4859748x+44287	0.01-1.0	1	—
Pea seeds	y= 3449788x+88064		0.9971	−29.0

Note: Matrix effect (%) = $(\frac{S_m}{S_s}-1)\times 100\%$; S_m is the slope of the calibration curve in the matrix (pea seeds); S_s is the slope of the calibration curve in the solvent (acetonitrile).

Table S4 Recoveries of acetamiprid in pea seeds (n = 5)

Spiked level (mg/kg)	Average recovery (%)	RSD (%)
2	96	7.4
1	93	3.7
0.2	82	7.7
0.01	79	11.6

Table S5 Gradient elution program of mobile phase for analyzing plant hormones

Time (min)	Phase A (%)	Phase B (%)	Flow rate (mL/min)
0.00	95.00	5.00	0.200
3.00	0.00	100.00	0.200
4.00	0.00	100.00	0.200
4.01	95.00	5.00	0.200
5.30	95.00	5.00	0.200

Note: phase A is a 0.1% (v/v) formic acid aqueous solution; phase B is acetonitrile.

Table S6 Mass parameters for analyzing plant hormones

Metabolite	Retention time (min)	Parent ion (m/z)	Daughter ion (m/z)	Cone voltage (V)	Collision energy (eV)	Polarity
IAA	1.70	174.1	130.1	50	5	−
SA	1.75	137.1	93.1	55	15	−
ABA	1.77	263.3	153.1	60	5	−
JA	2.00	209.2	59.1	75	5	−

Table S7 Gradient elution program of mobile phase for analyzing 4-hydroxybenzoic acid, caffeic acid, sinapic acid and ferulic acid

Time (min)	Phase A (%)	Phase B (%)	Flow rate (mL/min)
0.00	95.00	5.00	0.200
5.00	0.00	100.00	0.200
6.00	0.00	100.00	0.200
6.01	95.00	5.00	0.200
7.30	95.00	5.00	0.200

Note: phase A is 0.1% (v/v) formic acid aqueous solution; phase B is acetonitrile.

Table S8 Mass parameters for analyzing 4-hydroxybenzoic acid, caffeic acid sinapic acid and ferulic acid

Metabolite	Retention time (min)	Parent ion (m/z)	Daughter ion (m/z)	Cone voltage (V)	Collision energy (eV)	Polarity
4-Hydroxybenzoic acid	1.48	137.0	93.1	70	15	−
Caffeic acid	1.70	179.0	135.0	65	15	−
Sinapic acid	2.00	223.1	208.0	90	10	−
Ferulic acid	2.04	193.0	134.0	90	18	−

Table S9 Gradient elution program of mobile phase for analyzing quercetin, luteolin and kaempferol

Time (min)	Phase A (%)	Phase B (%)	Flow rate (mL/min)
0.00	95.00	5.00	0.200
4.00	0.00	100.00	0.200
5.00	0.00	100.00	0.200
5.01	95.00	5.00	0.200
6.30	95.00	5.00	0.200

Note: phase A is 0.1% (v/v) formic acid aqueous solution; phase B is acetonitrile.

Table S10 Mass parameters for analyzing quercetin, luteolin and kaempferol

Metabolite	Retention time (min)	Parent ion (m/z)	Daughter ion (m/z)	Cone voltage (V)	Collision energy (eV)	Polarity
Quercetin	1.72	301.0	151.0	140	20	−
Luteolin	2.12	285.0	133.0	160	35	−
Kaempferol	2.42	285.0	117.0	155	45	−

Table S11 Gradient elution program of mobile phase for analyzing phenylalanine, coniferyl alcohol and 4-coumaraldehyde

Time (min)	Phase A (%)	Phase B (%)	Flow rate (mL/min)
0.00	5.00	95.00	0.200
3.00	95.00	5.00	0.200
3.10	5.00	95.00	0.200
4.01	5.00	95.00	0.200

Note: phase A is 0.1% (v/v) formic acid aqueous solution; phase B is acetonitrile.

Table S12 Mass parameters for analyzing phenylalanine, coniferyl alcohol and 4-coumaraldehyde

Metabolite	Retention time (min)	Parent ion (m/z)	Daughter ion (m/z)	Cone voltage (V)	Collision energy (eV)	Polarity
Phenylalanine	0.79	180.1	163.1/120.0*	90	10/10	+
Coniferyl alcohol	0.82	179.1	119.0/91.1*	90	20/30	+
4-Coumaraldehyde	2.21	149.1	131.0/103*	90	10/20	+

Note: * indicates quantitative ions

Table S13 Gradient elution program of mobile phase for analyzing 4-coumaric acid, 4-coumaryl alcohol, sinapyl alcohol and caffeyl alcohol

Time (min)	Phase A (%)	Phase B (%)	Flow rate (mL/min)
0.00	5.00	95.00	0.200
3.00	95.00	5.00	0.200
3.01	5.00	95.00	0.200
4.00	5.00	95.00	0.200

Note: phase A is 0.1% (v/v) formic acid aqueous solution; phase B is acetonitrile.

Table S14 Mass parameters for analyzing 4-coumaric acid, 4-coumaryl alcohol, sinapyl alcohol and caffeyl alcohol

Metabolite	Retention time (min)	Parent ion (m/z)	Daughter ion (m/z)	Cone voltage (V)	Collision energy (eV)	Polarity
4-Coumaric acid	0.75	163.1	119.0	50	20	−
4-Coumaryl alcohol	2.33	151.0	128.1/110.0*	60	4/4	+
Sinapyl alcohol	2.35	153.1	119.9/67.7*	60	4/10	+
Caffeyl alcohol	2.37	167.2	126.0/84.9*	60	4/10	+

Note: * indicates quantitative ions
